# A Clinical Case Report of a Potential Acute Allergic Reaction with Titanium Dental Implant

**DOI:** 10.1155/2021/5592934

**Published:** 2021-04-10

**Authors:** Ali Robaian Alqahtani, Khalid Gufran, Fred Silva, Mateus Garcia Rocha, Jia Chang

**Affiliations:** ^1^Department of Conservative Dental Sciences, College of Dentistry, Prince Sattam Bin Abdulaziz University, Alkharj, Saudi Arabia; ^2^Department of Preventive Dental Sciences, College of Dentistry, Prince Sattam Bin Abdulaziz University, Alkharj, Saudi Arabia; ^3^Department of Periodontics, Texas A & M College of Dentistry, Dallas, TX 75246, USA; ^4^Center for Dental Biomaterials, Division of Operative Dentistry, Department of Restorative Dental Sciences, College of Dentistry University of Florida, USA; ^5^Department of Periodontology, College of Dentistry, University of Florida, USA

## Abstract

Titanium hypersensitivity is rare, but it may exist among patients who need dental implant treatment. It could contribute to mild or severe peri-implant tissue hypersensitivity reactions and affect patients' oral and general health according to some clinical reports. In extreme cases, it may lead to implant failure or extraction. In this case report, a 64-year-old Caucasian female patient received a titanium implant placement on #5. Right after the implant placement, she reported the symptoms of pain, eczema, and slight extraoral swelling, along with significant burning sensation occurring intra- and extraorally. The symptoms were not released after the systemic intervention of antibiotics for six days. On retrieving her medical history, the patient reported a previous allergic reaction to jewelry-like earrings in her childhood. The diagnosis of titanium hypersensitivity was made based on the rapid onset of symptoms and her metal allergy history. Therefore, the dental implant was removed after diagnosis, and a bone allograft was used to preserve the alveolar bone volume. Immediately after implant extraction, the swelling and burning sensation were receded. A complete recovery was achieved three weeks later. The dimension of the alveolar ridge bone was found well maintained in 10 weeks follow-up visit. *Conclusion*. Rapid onset of peri-implant gingival swelling may indicate a hypersensitivity to titanium implant in the clinic. For patients with a history of allergy to jewelry, the hypersensitivity tests to titanium alloy such as patch test or lymphocyte transformation test should be recommended as part of the dental implant treatment plan.

## 1. Introduction

Titanium (Ti) and its alloys have been widely used as dental implant material due to high biocompatibility, resistance to corrosion, and mechanical properties [1–3]. However, some clinicians reported their patients may show hypersensitivity to medical or dental titanium implants, which is demonstrated by a temporal association between exposure to titanium and occurrence of tissue reaction proximal to implanted titanium [4–10]. For example, Sicilia et al. reported approximately 0.6% of patients receiving titanium dental implant treatment presented a positive reaction to titanium in the epicutaneous tests [6]. Egusa et al. reported an implant failure case due to suspected severe titanium allergy. The patient exhibited facial eczema following the placement of mandibular titanium dental implants for supporting overdenture. The removal of the implants resulted in a complete remission of symptoms [5]. Pigatto et al. reported a clinical case of exfoliative cheilitis correlated to a titanium dental implant placement in a 41-year-old female [11]. Clinically, the oral manifestations of hypersensitivity to dental materials and dental implant may present as mucosal erythema, swelling of lips or purpuric patches on hard and soft palate, ulcers in oral cavity, hyperplastic gingivitis, tongue depapillation, angular cheilitis, eczematous eruptions on the face, or lichenoid reactions, etc. [12].

Allergic reactions include immediate humoral response due to antibody/antigen complexes in type I, II, III reactions, or type IV delayed hypersensitivity. In oral cavity, titanium implant-related allergic reactions usually occurred as type IV hypersensitivity reactions [13, 14]. The mechanism of such allergic reactions is commonly related to tribocorrosion, which is a material degradation process due to the combined effect of wear and corrosion. Mechanical wear occurs during implant placement and loading. Corrosion occurs once the oxide layer of titanium implants is disturbed after titanium exposed to fluid media or air. Any disruption of the oxide surface layer will cause corrosion and compromise implant biocompatibility [13]. Mechanical wear and corrosion can generate metal debris or particles from titanium or its alloy components. In animal studies, researchers found the increased presence of titanium ions in peri-implant tissues following implant placement and detected titanium ions in regional lymph nodes and the lungs [15–17]. These metal ions may bind with serum proteins and activate the immune system by inducing the production of specific T lymphocytes, which eventually resulting in type IV hypersensitivity reaction [18, 19]. Usually, an epicutaneous patch test is used to diagnose metal allergies in patients. However, its results could be a false positive reaction because the procedure itself may induce sensitivity or cause a flare-up of symptoms [20]. The lymphocyte transformation test (LTT) becomes a more suitable test to assist in diagnosing metal hypersensitivity, including titanium materials [21, 22]. Its predictive value needs more studies in order to be proven [4].

In this case report, we exhibited a female patient's acute allergic reaction to a titanium implant placed in her maxillary premolar area. Her initial symptoms of hypersensitivity reaction, response to antibiotics intervention, medical history retrieval, implant extraction, and tissue healing outcomes in the short-term and long-term follow-up were recorded. We aim to introduce the process of our clinical diagnosis and management of dental implant hypersensitivity, as well as addressing the importance of retrieving a patient's hypersensitivity history and performing a hypersensitivity test during implant treatment planning for patients with a history of hypersensitivity to jewelry or dental materials.

## 2. Case Presentation

**Figure 1:**
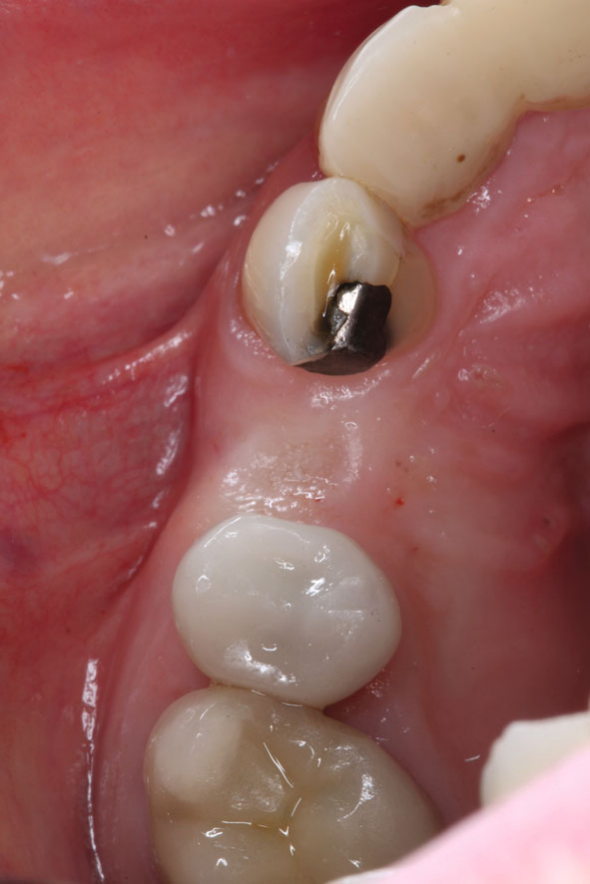
Preimplant surgery photo.

**Figure 2:**
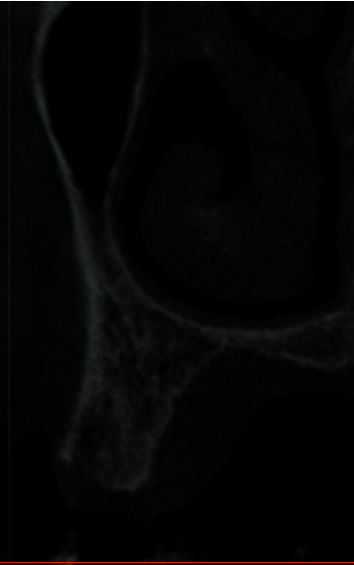
CBCT image before implant placement.

**Figure 3:**
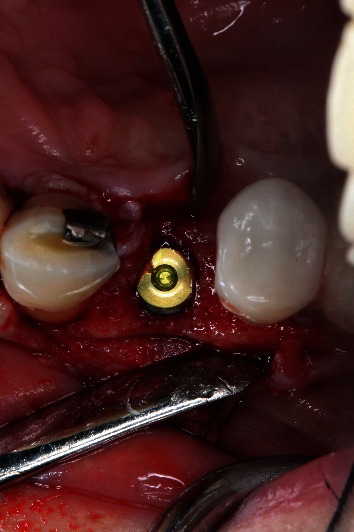
Implant placement.

**Figure 4:**
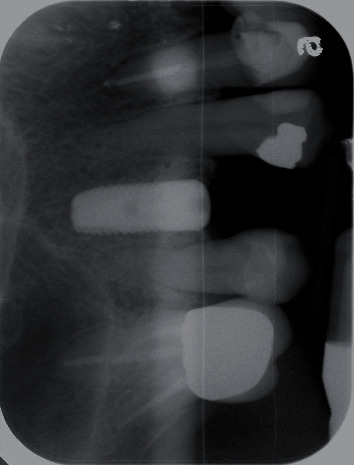
X-ray following the implant placement.

**Figure 5:**
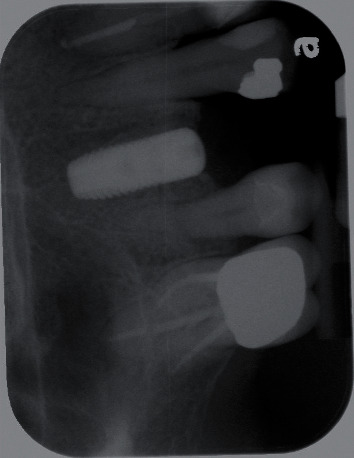
10 days after implant placement. The patient exhibited significant allergy symptoms.

**Figure 6:**
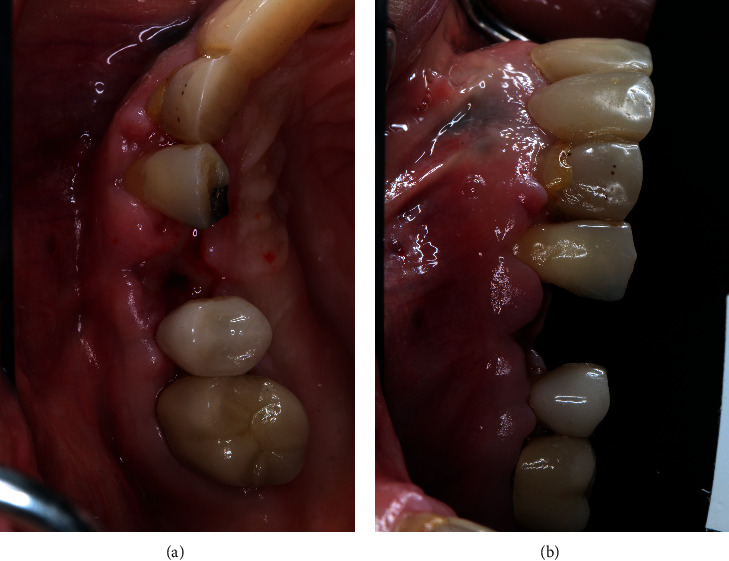
Intraoral photos taken on day 4 after implant placement exhibit significant allergy symptoms. ((a) occlusal view; (b) buccal view).

**Figure 7:**
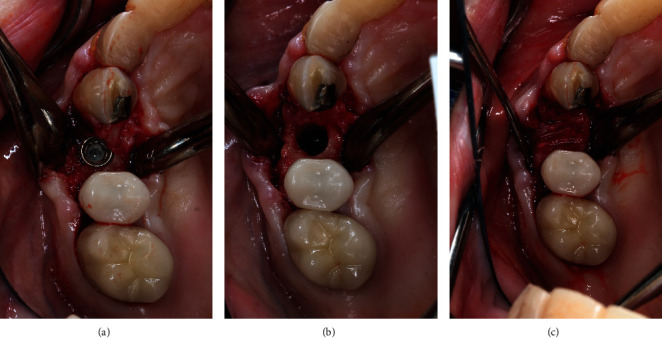
Implant removal surgery. (a) Implant exposure. (b) Implant removal. (c) Bone grafting of implant site.

**Figure 8:**
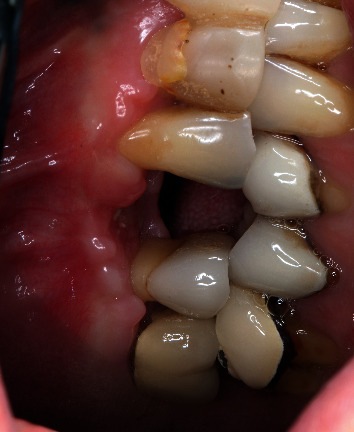
Three-week postsurgical follow-up.

**Figure 9:**
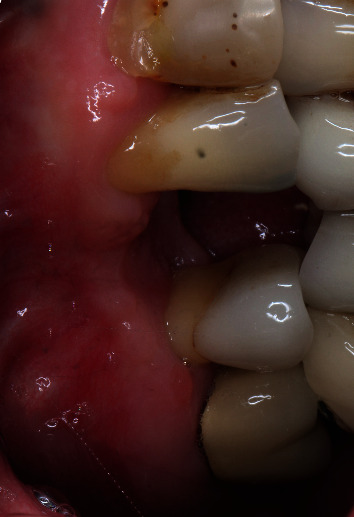
Ten-week postsurgical follow-up.

A 64-year-old Caucasian female patient was referred to the Graduate Periodontics Clinic at the University of Florida College of Dentistry for implant placement in the area of #5 (Figure 1). In the patient's self-report about her medical history, she has known allergies to Codeine/Narcotics and some food products, along with hypertension and hypothyroidism. All of her medical conditions were well monitored and controlled by medications. The extraoral and intraoral examinations were performed, including a full periodontal charting examination, periapical radiography, and cone-beam computed tomography. The implant treatment plan was made after assessing the bone volume as well as its proximity to the vital structures in #5 site (Figure 2). After obtaining the patient's consent, the surgical implant placement was performed at #5 area (4.2 mm × 10 mm Astra Tech EV OsseoSpeed, Dentsply International) (Figures 3 and 4). The placed implant achieved good primary stability. The cover screw was tightened, and the site was sutured using a 4-0 chromic gut absorbable suture. The surgery was completed smoothly. The surgical wound showed no bleeding and swelling after suturing. The patient took Ibuprofen for postsurgical pain management. Four days after implant surgery, the patient called the surgeon and complained of the progressing pain, swelling, and burning sensation intraorally and extraorally on her implant surgery side. The patient was scheduled for a follow-up appointment immediately. In the follow-up visit, this patient claimed that from 2^nd^ day after the implant placement, she felt mild to moderate pain at the implant surgery site, which gradually became severe with a score of 8 out of 10 on the pain scale. There was mild swelling with redness on her right side of the face. No significant lymphadenopathy in the orofacial and neck regions was found. The intraoral examination showed erythema of the oral mucosa around the surgical site with purpuric patches on the palate along with few mouth ulcers. The surgical site and adjacent teeth were tender to palpation with hyperplastic gingival (Figure 5). A periapical radiograph was taken during the appointment, and no apparent abnormality was detected (Figure 6). The patient was reassured, and a prescription of amoxicillin (500 mg every 8 hours for seven days by oral administration) was given. The patient texted the surgeon six days later, claiming the symptoms had not been improved since her last visit. Besides, she wrote, “My mother called and reminded me I am allergic to certain metals. For example, I cannot wear some earrings; my ear lobe swells up then bleeds. Do you think this could be the case with this implant?” Based on the patient's history of metal hypersensitivity and her postimplant surgery symptoms, we highly suspected she was allergic to the newly-placed titanium implant on #5. The patient was immediately scheduled for the explant surgery, and the implant on #5 was removed (Figures 7(a) and 7(b)). The extracted site was grafted with freeze-dried bone allograft (Symbios allograft, Dentsply Sirona) and covered with resorbable collagen membrane (Symbios Collagen Membrane pre-hydrated, Dentsply Sirona) (Figure 7(c)). The wound was closed using 3-0 PTFE nonabsorbable suture material. The patient was instructed to continue with her medication. The patient reported that the facial and gum swelling, the burning sensation, and severe pain had subsided soon after the surgery. The patient had 3, 6, and 10-week follow-up visits (Figures 8 and 9). The surgical wound healed very well after the surgery. The ridge bone volume on the site was well-maintained. The patient still interests in implant-supported crown restoration. She will be scheduled for a hypersensitivity test to have the diagnostic analysis. It may help to determine whether she is indicated for zirconium implant in the future.

## 3. Discussion and Conclusions

In this case report, the patient received titanium dental implant surgery and exhibited pain, eczema, slight extraoral swelling, and burning sensation intra- and extraorally within four days after the implant placement. The symptoms got worse and spread to the broader orofacial area even with antibiotic administration, which, in this case, excludes bacterial infection as the etiology. A history of allergy to metal earrings was retrieved according to her communication with her mother. Based on her previous dental treatment record, we excluded possible allergic reaction to chromic gut absorbable suture placed during her implant surgery and ibuprofen medication for pain management after the implant surgery. Therefore, the initial diagnosis of titanium hypersensitivity was highly suspected. To manage her significant tissue reactions, the titanium implant was extracted, and the drilling site was grafted with allograft bone materials. All intra- and extraoral symptoms receded right after the implant removal, and a complete recovery was achieved three weeks later. Such responses confirmed the initial clinical diagnosis of hypersensitivity to titanium-based dental implants. The patient was referred to an immunology center to have a medical consult about potential allergens before implant treatment plan in the future.

There are 3 types of allergic reactions related to the orofacial region—type I, III, and IV. While type I is an immediate anaphylactic reaction to allergens, type III can occur 2–8 hours after the placement of implants due to the increase in circulatory antibodies. However, it may appear even after fourteen days. Type IV is a delayed reaction, which occurs 24–72 hours after the surgery, and it occurs after recurrent contact of the allergen with the mucosa or skin, but the symptoms may appear up to 14 days postimplant placement. Allergic reaction could appear at some distance from the implant and can cause a systemic reaction, which could be overlooked or misinterpreted [23].

The rapid onset of hypersensitivity reaction proximal to the inserted dental implant as shown in this case report is extremely rare, and most of titanium hypersensitivity reactions are of delayed type [24].

Titanium alloys are preferred as dental implants when compared to pure titanium, owing to their superior strength. It is worth noting that even pure titanium is not free of trace impurities such as aluminum, manganese, iron, beryllium, and nickel, which can cause an allergic reaction. Allergic reaction to metals including titanium occurs due to the ions produced as a result of implant corrosion, which could either come in contact with the mucosa or could be ingested. Although these ions by themselves are not irritating, they can combine with native proteins to form complexes, which could be a potential allergen-inducing hypersensitive reaction [23].

Since titanium is considered a gold standard for dental implants, an allergic reaction is not expected by dental professionals. Literature contains very limited data regarding the incidence and management of allergic reaction arising from titanium dental implants. Therefore, improved guidelines are warranted before the placement of an implant, and if an allergic reaction does occur post implant placement, all possible treatment alternatives must be explained and made available. Carrying out titanium allergy test should be routinely followed, especially in patients with known allergies to metals. If the patient experiences hypersensitive reaction following dental implant placement, the possibility of explanation should be considered depending on the risks and benefits for the patient. Zirconium dioxide implants (ZrO2) or yttria-stabilized zirconia can serve as good substitutes as no allergic reactions have been reported yet from zirconium oxide [25]. It can be concluded that although rare, titanium hypersensitivity is a real possibility and should not be overlooked since an allergic reaction could cause various symptoms.

## Data Availability

The data that support the findings of this case report are available from the corresponding author upon reasonable request. All surgical photos are presented in the figures of this submission. The patient's treatment history and records are documented and saved in the Axium electronic patient recording system of the University of Florida, College of Dentistry.

## Abbreviations

Ti: Titanium
LTT: Lymphocyte transformation tests.
